# An unusual cause of dysphagia in ductal breast cancer due to submucosal oropharyngeal metastatic spread: a case report

**DOI:** 10.1186/1757-1626-2-3

**Published:** 2009-01-02

**Authors:** Dorothy M Gujral, Mara Quante, Richard AJ Simcock

**Affiliations:** 1 Sussex Cancer Centre, Royal Sussex County Hospital, Eastern Rd, Brighton, BN2 5BE, UK; 2 Department of Pathology, Royal Sussex County Hospital, Eastern Rd, Brighton, BN2 5BE, UK

## Abstract

**Introduction:**

Invasive ductal and lobular carcinomas represent 67.9% and 6.3% of breast carcinoma, respectively. Metastatic breast cancer typically involves the lungs, bones, brain, and liver. Studies have shown differing patterns of metastatic spread between ductal and lobular carcinoma. Lobular carcinoma is more likely to metastasise to the gastrointestinal tract.

**Case presentation:**

We report the case of a 49 year old white woman with invasive ductal carcinoma with lobular differentiation who developed submucosal oropharyngeal metastases nearly two years after her original diagnosis after presenting with odynophagia and dysphagia. The patient's symptoms preceded any associated radiological or endoscopic abnormalities by at least 9 months. Repeat computed tomography scan and eventual oropharyngeal biopsy confirmed submucosal metastatic invasive ductal carcinoma, suggesting occult submucosal spread.

**Conclusion:**

This case illustrates the importance of maintaining a high index of suspicion for metastatic disease in patients with invasive breast cancer who present with unusual symptoms and a careful search for metastatic sites.

## Introduction

Invasive ductal and lobular carcinomas represent 67.9% and 6.3% of breast carcinoma, respectively [1]. Metastatic breast cancer typically involves the lungs, bones, brain, and liver [2]. Studies have shown differing patterns of metastatic spread between ductal and lobular carcinoma [3]. Borst and Ingold [4] reported a significantly higher prevalence of metastatic lobular cancer to the gastrointestinal (GI) tract, peritoneum and retroperitoneum, and gynaecological organs, than metastatic ductal cancer. The most common sites of gastrointestinal involvement are the stomach and colon [4].

## Case presentation

A 49 year old white woman was diagnosed in August 2005 with a grade 2 invasive mixed ductal and lobular carcinoma of the left breast (oestrogen receptor negative, progesterone receptor negative, HER2 receptor negative) with left supraclavicular lymphadenopathy, but no axillary lymphadenopathy.

The patient received 6 cycles of neoadjuvant epirubicin, cyclophosphamide and 5-fluorouracil (FEC) chemotherapy with good response. In February 2006 she was found to have two enlarged right supraclavicular lymph nodes. Planned surgery was therefore abandoned and the patient proceeded to weekly paclitaxel chemotherapy, receiving 6 cycles before progressing with new nodes in both supraclavicular fossae and left cervical lymph nodes and difficulty swallowing. The patient also complained of discomfort in the anterior neck. Both radiological and ultrasonographic examinations were normal.

The patient received radiotherapy to the left breast, axilla and left supraclavicular fossa (40 Gray in fifteen fractions to the left breast and 50 Gray in twenty-five fractions to the left axilla and supraclavicular fossa) followed by gemcitabine and carboplatin chemotherapy with good response – staging computed tomography (CT) scan showed good response to chemotherapy with no significantly enlarged lymph nodes.

In view of ongoing dysphagia/odynophagia and the earlier presentation with supraclavicular lymphadenopathy, the patient underwent endoscopy which revealed mild gastritis. She completed a ten day course of oral fluconazole and was prescribed maintenance proton pump inhibitor. CT scans were repeated 7 months later due to persistent symptoms of odynophagia. This scan confirmed stable disease with some generalised thickening of the oesophagus in the subcarinal region in relation to small volume adenopathy; however, this was unchanged compared to previous imaging. Barium swallow showed no hold-up, stricturing or dysmotility. There was no mucosal abnormality with no evidence of a cricoid web. This was confirmed on repeat endoscopy.

**Figure 1:**
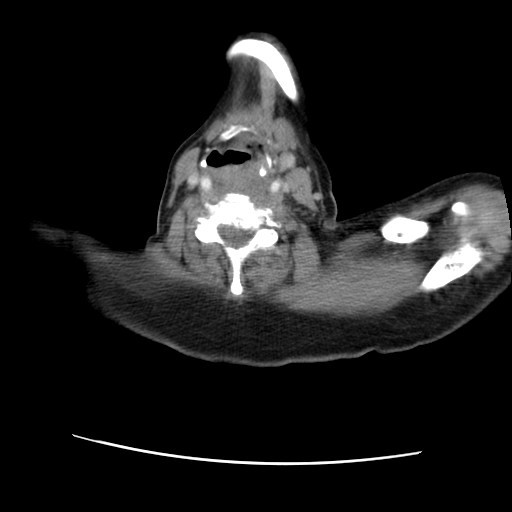
**CT slice showing new lesion in oropharynx**.

**Figure 2:**
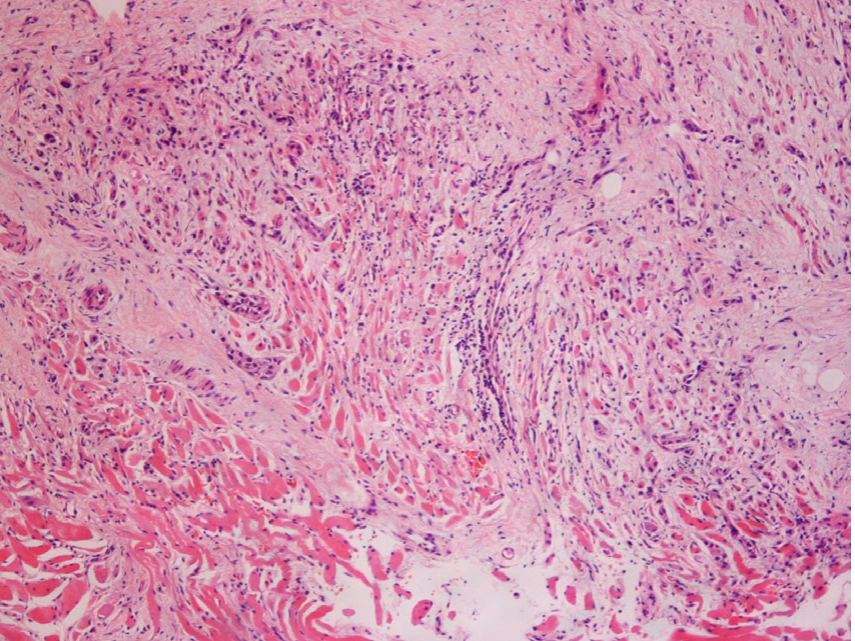
**Biopsy of oropharnyx confirming submucosal metastatic invasive ductal carcinoma**.

**Figure 3:**
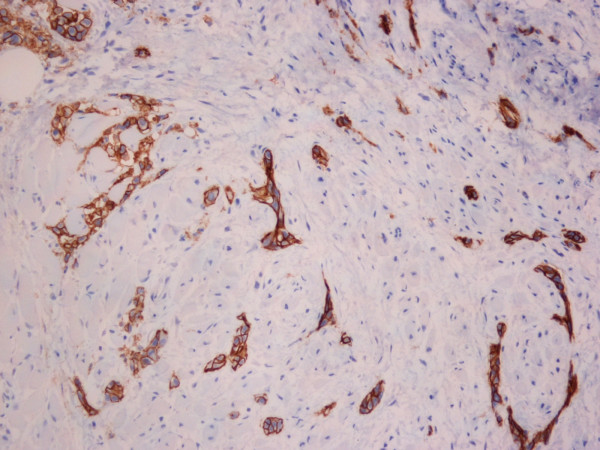
**E-cadherin stain on oropharyngeal biopsy**.

As a result of ongoing odynophagia, a repeat CT scan was requested at a further 2 month interval (nine months after the onset of symptoms) which revealed a new lesion in the oropharynx [fig 1] and significant adenopathy within the neck (cervical and supraclavicular lymph nodes), more marked on the left than right. There was also note made of some soft tissue thickening in the prevertebral space below the level of the larynx and circumferential thickening of the cervical and proximal thoracic oesophagus with no discrete mass. Fibre-optic nasendoscopy revealed no asymmetry of the oropharynx. Subsequent examination under anaesthetic and biopsy of the area confirmed submucosal metastatic invasive ductal carcinoma with some lobular differentiation [fig 2]. E-Cadherin staining showed strong expression within the majority of the tumour cells [fig 3]. E-Cadherin stain was then performed on the original biopsy and showed strong expression, supporting the diagnosis of an original ductal carcinoma with lobular features, rather than a pure lobular carcinoma.

The patient had a radiologically-inserted gastrostomy tube inserted for feeding and commenced palliative radiotherapy to the neck to cover the oropharyngeal disease but her disease progressed during treatment with airway narrowing and stridor. The patient is currently being palliated at home.

## Discussion

Breast cancer metastases to the gastrointestinal tract (GIT) is rare, and is more frequently a feature of lobular infiltrating carcinoma [5]. The pattern of spread and infiltration by lymphatics to the oesophagus typically results in stricture formation or submucosal formation of the metastatic tumour mass with normal overlying mucosa, thereby making endoscopic diagnosis difficult [6-8]. The patient's symptoms preceded any associated radiological or endoscopic abnormalities by at least 9 months suggesting occult submucosal spread. The absence of stricturing on barium study is an unusual feature of this case. In addition, oropharyngeal spread is less reported. In a recent review of the literature by Nazareno et al [9] one case of oropharyngeal metastasis was described and this was a discrete mass in the post cricoid region rather than submucosal spread as illustrated in this case. Raut et al [10] described a case of metastatic adenocarcinoma of the breast as a discrete mass in the parapharyngeal space, fifteen years after the primary diagnosis. Just et al [11] report a case of a woman with breast cancer who developed papillomatosis and acanthosis of the oral cavity prior to the patient developing metastatic disease of the liver, which was followed by regression of the oral lesions after treatment of the liver metastases, in keeping with a paraneoplastic phenomenon.

About 50% of all metastatic disease of the oesophagus present with dysphagia [12] and metastatic breast carcinoma has been estimated to account for 0.4% of all cases of symptomatic oesophageal obstruction [13]. The onset of dysphagia secondary to oesophageal metastasis from primary diagnosis is typically long, with a mean time of mastectomy to the onset of dysphagia of 7.1 +/- 4.2 years with a peak incidence of 4 to 5 years [14].

The reason for these differences in the metastatic patterns of lobular carcinoma and infiltrating ductal carcinoma is unknown. Invasive ductal carcinoma (IDC) and invasive lobular carcinoma (ILC) have distinct histological patterns. IDC are composed of cells arranged in well formed glandular structures, whereas ILC correspond to a proliferation of non-cohesive small cells dispersed in a fibrous stroma [15]. It has been suggested that loss of expression of the cell-cell adhesion molecule E-cadherin in infiltrating lobular carcinoma could result in the non-cohesive tumour cells and this may play a part in the differing pattern of local and metastatic tumour progression [16]. Down-regulation of E-cadherin may facilitate cell spreading and/or cell growth, resulting in tumour growth and dissemination. It is therefore even more unusual that metastatic spread to uncommon sites should occur in patients with ductal carcinoma.

## Conclusion

This case illustrates the importance of maintaining a high index of suspicion for metastatic disease in patients with invasive breast cancer who present with unusual symptoms and a careful search for metastatic sites, even in those patients with ductal carcinoma. In this case, earlier imaging with a positron-emission tomography scan (PET scan) may have been useful, but would not have relinquished the need for endoscopy and tissue biopsy to confirm the diagnosis.

## Consent

Written consent was obtained from the patient for publication of this case report and accompanying images. A copy of the written consent is available for review by the Editor-in-Chief of this journal.

## Competing interests

The authors declare that they have no competing interests.

## Authors' contributions

DG and RS were involved in the clinical care of the patient. DG and RS conceived, researched, wrote the paper and revised the final manuscript. MQ prepared and interpreted the histological material. All authors read and approved the final manuscript
